# Characterization of Human CD4 T Cells Specific for a C-Peptide/C-Peptide Hybrid Insulin Peptide

**DOI:** 10.3389/fimmu.2021.668680

**Published:** 2021-05-25

**Authors:** Timothy A. Wiles, Anita Hohenstein, Laurie G. Landry, Mylinh Dang, Roger Powell, Perrin Guyer, Eddie A. James, Maki Nakayama, Kathryn Haskins, Thomas Delong, Rocky L. Baker

**Affiliations:** ^1^ Department of Pharmaceutical Sciences, University of Colorado Skaggs School of Pharmacy and Pharmaceutical Sciences, Aurora, CO, United States; ^2^ Department of Immunology and Microbiology, University of Colorado School of Medicine, Aurora, CO, United States; ^3^ Barbara Davis Center for Childhood Diabetes, University of Colorado School of Medicine, Aurora, CO, United States; ^4^ Department of Translational Research, Benaroya Research Institute at Virginia Mason, Seattle, WA, United States

**Keywords:** CD4, hybrid insulin peptides, autoimmunity, mass spectrometry, type 1 diabetes, neoepitope, tetramer

## Abstract

Hybrid Insulin Peptides (HIPs), which consist of insulin fragments fused to other peptides from β-cell secretory granule proteins, are CD4 T cell autoantigens in type 1 diabetes (T1D). We have studied HIPs and HIP-reactive CD4 T cells extensively in the context of the non-obese diabetic (NOD) mouse model of autoimmune diabetes and have shown that CD4 T cells specific for HIPs are major contributors to disease pathogenesis. Additionally, in the human context, HIP-reactive CD4 T cells can be found in the islets and peripheral blood of T1D patients. Here, we performed an in-depth characterization of the CD4 T cell response to a C-peptide/C-peptide HIP (HIP11) in human T1D. We identified the TCR expressed by the previously-reported HIP11-reactive CD4 T cell clone E2, which was isolated from the peripheral blood of a T1D patient, and determined that it recognizes HIP11 in the context of HLA-DQ2. We also identified a HIP11-specific TCR directly in the islets of a T1D donor and demonstrated that this TCR recognizes a different minimal epitope of HIP11 presented by HLA-DQ8. We generated and tested an HLA-DQ2 tetramer loaded with HIP11 that will enable direct *ex vivo* interrogation of CD4 T cell responses to HIP11 in human patients and control subjects. Using mass spectrometric analysis, we confirmed that HIP11 is present in human islets. This work represents an important step in characterizing the role of CD4 T cell responses to HIPs in human T1D.

## Introduction

Type 1 diabetes (T1D) is an autoimmune disease driven by T cells specific for pancreatic β-cell autoantigens. Identifying β-cell antigens targeted by autoreactive T cells is an important step in understanding the etiopathogenesis of T1D. Post-translational modification of proteins can generate neo-epitopes in the periphery that are not represented in the thymus, providing a mechanism by which autoreactive T cells can evade central tolerance mechanisms and mediate β-cell destruction in the pancreatic islets. Hybrid Insulin Peptide (HIP) formation is a recently discovered modification that may play a key role in the generation of primary targets of autoimmunity in T1D (1). HIPs are formed by covalent fusion of the N-terminus of β-cell secretory granule peptides (a right peptide) to the C-terminus of truncated insulin peptides (a left peptide) through a peptide bond. The resulting HIPs contain amino acid sequences not encoded by the genome, making them plausible targets for autoreactive T cells in T1D. We have identified HIPs in murine and human islets by mass spectrometry (1, 2), and a separate group recently demonstrated that HIPs can be found in the major histocompatibility complex (MHC) class II peptidome of mice (3).

In contrast to conventional post-translational modifications, such as phosphorylation or deamidation, which alter the side chain of a single amino acid residue, HIP formation leads to the replacement of an entire sequence of amino acids within insulin. This could have marked consequences for how a HIP, when compared to the unmodified precursor, binds to a given human leukocyte antigen (HLA) molecule and interacts with a specific T cell receptor (TCR). This, in turn, could result in recognition of a HIP in the periphery by T cells that did not recognize the corresponding unmodified peptides in the thymus during negative selection.

The role of HIPs as autoantigens in the non-obese diabetic (NOD) mouse model of T1D is clearly established. We have demonstrated that HIPs are the peptide ligands of diabetogenic CD4 T cell clones isolated from NOD mice (1). HIP-specific CD4 T cells display an inflammatory phenotype and can be detected in the pancreatic lymph nodes (pLN), islets, and spleen of prediabetic and diabetic NOD mice using MHC class II tetramers (4). The frequency of HIP-specific CD4 T cells in the blood of NOD mice increases with age, as do the percentages of HIP-specific CD4 T cells that display an activated phenotype, suggesting that these cells may serve as biomarkers of disease progression (4).

Evidence supporting an important role for HIPs as autoantigens in human T1D is also mounting. Multiple CD4 T cell clones/lines specific for different HIPs have been isolated from the residual islets of human organ donors with T1D (1, 5–7). As recently reported, we screened peripheral blood mononuclear cells (PBMCs) from T1D patients or controls for reactivity to a panel of HIPs by IFN-γ enzyme-linked immunosorbent spot (ELISpot) analysis (6). We observed statistically significant responses from patient PBMC to four of the HIPs tested. One of those HIPs (HIP11) is a hybrid peptide formed by the fusion of a C-peptide fragment with the N-terminus of C-peptide. Robust responses to HIP11 were detected in samples from more than 20% of the 35 patients tested (6). Here, using the CD4 T cell clone E2 isolated from the PBMCs of a new onset T1D patient (6), we describe the further characterization of the CD4 T cell response to HIP11, advancing our understanding of HIPs as autoantigens in T1D and providing a foundation for future efforts to investigate responses to HIPs during the development of T1D.

## Materials and Methods

### Peptides

The list of peptides used in this study were obtained commercially at >95% chromatographic purity and are listed in **Supplementary Table 1**.

### HIP Nomenclature

To avoid ambiguity and confusion, we developed a systematic nomenclature to describe the large diversity of potential HIP sequences. HIPs are identified using standard abbreviations for the proteins from which the left and right peptides originated (*e.g*., Ins, IAPP, CgA), separated by a dash. Next to each abbreviation, a subscript is used to describe the residues from the parent protein that span the sequence. For easy reference, insulin peptides are designated as “insA”, “insB”, or “insC”, indicating that the peptide originated from the A-chain, B-chain, or C-peptide region of insulin, respectively, and numbering is based on the position of the residues within the specific peptide. For peptides from all other proteins, residues are numbered based on their position in the pre-proprotein, with position 1 being the N-terminal residue of the signal peptide. For example, the HIP sequences DLQVGQVELGGGPGAGSLQPLAL-EAE and SLQPLAL–EAEDLQV (hyphen indicates the HIP junction) referenced in this study are designated insC_4-26_ – insC_1-3_ and insC_20-26_ – insC_1-7_, respectively.

The defining feature of any individual HIP is its junction region. Trimming of a HIP – as may occur within an antigen-presenting cell, the proteasome, or with a protease used during sample preparation prior to LC-MS/MS analysis – could lead to the generation of peptides of different lengths that all represent the same HIP (*i.e.*, they contain the same junction). Common names can be used to refer to a specific HIP junction. For example, the HIP generated when an insulin C-peptide fragment ending with residue 26 is fused to the N-terminus of C-peptide (insC_x-26_ – insC_1-x_) has been designated HIP11 (1). Both insC_4-26_ – insC_1-3_ and insC_20-26_ – insC_1-7_ are considered HIP11 peptides.

### Sequencing and Generation of E2a and E2b Transductants

TCRα and TCRβ chain genes were amplified by 5’RACE-PCR, followed by sequencing on an MiSEQ sequencer (Illumina) as previously described (6). V- and J-gene usages as well as CDR3 sequences were then analyzed by IMGT/V-QUEST (http://www.imgt.org). The determined TCRα and TCRβ chain genes were expressed on a 5KC hybridoma cell line transduced with the NFAT-ZsGreen-1 reporter construct using a retroviral expression system as previously described (8). Briefly, a murine stem cell virus-based retroviral vector encoding TCRα and TCRβ chain genes connected by a porcine-2A peptide was generated, and the reporter 5KC cell line was spinfected with a virus supernatant, followed by MACS-based enrichment of transduced cells using magnetic beads conjugated with anti-CD3 antibody. The E2a and E2b transductants (2 x 10^4^ cells/well) were cultured with or without peptides along with K562 cells expressing DQ2 (DQA1*05:01-DQB1*02:01) or DR3 (DRA1*01:01-DRB1*03:01) at 5 x 10^4^ cells/well in a round-bottom 96 well plate overnight, followed by evaluating ZsGreen-1 expression by the TCR transductants on a Cytoflex flow-cytometer (Beckman Coulter).

### GSE.8E3 Transductant Activation Assay

GSE.8E3 transductant cells (2 x 10^4^ cells/well), produced by the same method used to generate the E2a and E2b transductants, were cultured with various concentrations of peptides in the presence of K562 cells expressing DQ8 (DQA1*03:01-DQB1*03:02) or DQ8*trans* (DQA1*05:01-DQB1*03:02) at 5 x 10^4^ cells/well in a round-bottom 96 well plate overnight. ZsGreen-1 expression by the TCR transductants was analyzed on a Cytoflex flow-cytometer (Beckman Coulter).

### T Cell Clone Assays

Early-passage CD4 T cell clones (E2, a HIP11-reactive clone and B11b, a HIP4-reactive clone) were thawed and restimulated with HIP11 or HIP4, respectively, in the presence of irradiated autologous EBV-transformed B-cell line as antigen-presenting cells (APC). Twenty-four hours later, IL-2 and IL-4 were added to the T cell cultures for T cell expansion. T cells were maintained in culture for 14-28 days before functional assays were performed. For T cell assays, CD4 T cell clones (0.5 – 5 x 10^5^) were incubated either with the irradiated autologous EBV-transformed B-cell line (0.5 – 5 x 10^5^ cells) or with irradiated PBMC from a partially HLA matched donor, in the presence or absence of antigen at indicated concentrations. For IFN-γ and TNF-α ELISA, supernatants were collected 24-48 hours after culture and analyzed using a kit from eBiosciences according to manufacturer’s protocol. To determine the DR- or DQ-HLA restriction of HIP responses, the antigen assay was performed in the presence and absence of anti-DR (L243) or anti-DQ (SPV-L3) antibodies. The EBV-transformed cell lines were first pulsed with peptide (50 μg/ml) and washed twice with AIM V medium. Antibodies were added at a final concentration of 1 μg/ml.

### Flow Cytometry

For the CD25 upregulation assay, 1 x 10^5^ CD4 T cells were cultured with an autologous EBV-transformed B cell line (1 x 10^5^ cells) in the presence or absence of peptide. After 24 hours, cells were harvested, washed, and stained for CD4, CD25 and viability (efluor780, Invitrogen) before analysis. Antibodies used for staining of T cells were anti-CD4 BV711 (SK3; BD Biosciences) and anti-CD25 BV421 (M-A251; BD Biosciences). Gating strategies are indicated in each figure; the lymphocyte gate was based on FSC/SSC properties and the singlets gate was based on the FSC-A/FSC-H. Samples were run on a BD Fortessa X-20 flow cytometer (5 lasers), a Cytek Aurora (5 lasers) or a CytoFlex (Beckton Dickinson) flow cytometer (3 lasers), and 5,000 – 50,000 cells were acquired for analysis. Data were analyzed using FlowJo v10 software (Tree Star, USA).

### Preparation of Islet Proteins

Islets from a non-diabetic human donor (64 year-old male) were obtained from the University of Alberta Diabetes Institute Islet Core (Edmonton, Alberta, Canada). Upon arrival, islets were washed with phosphate-buffered saline (PBS) and then pelleted. The supernatant was removed and tubes were placed in liquid nitrogen to rapidly freeze the islet pellets. Islets were stored at -80°C until use. Islets were processed as described previously (2). Briefly, islets were thawed and lysed in 50% trifluoroethanol with heat and sonication, and cellular debris was pelleted by high-speed centrifugation. The supernatant, which contained extracted islet proteins, was fractionated by size exclusion chromatography (SEC). SEC fractions were then digested overnight at 37°C with the endoproteinase AspN (cleaves at the N-terminal side of aspartic acid residues) in buffer supplemented with zinc sulfate. Digested samples were dried in a vacuum concentrator and then reconstituted in loading buffer (2.7% acetonitrile/0.1% formic acid/water), sonicated, and spun at 17,000 x g for 2 minutes to remove any insoluble material. The supernatant was then analyzed by liquid chromatography tandem-mass spectrometry (LC-MS/MS).

### LC-MS/MS Analysis of Human Islet Proteins

Samples were analyzed by LC-MS/MS using an Agilent 1200 series UHPLC system with a nanoflow adapter and an Agilent 6550 Q-TOF equipped with a nano-ESI source. Samples were separated online by reversed-phase liquid chromatography using a trap forward-elute configuration (trap column: Thermo Acclaim Pepmap 100, 75 µm x 2 cm, 3 µm particles, 100Å pores; analytical column: Thermo Acclaim Pepmap RSLC C18 analytical column, 75 µm inner diameter, 2 µm particles, 100Å pores). A water/acetonitrile gradient was used (buffer A: 0.1% formic acid in water; buffer B: 0.1% formic acid and 90% acetonitrile in water). Mass spectrometric data was collected in positive ion mode with an MS scan range of 290-1700 m/z, an MS acquisition rate of 5 spectra/sec, an MS/MS scan range of 50-1700 m/z, and a minimum MS/MS scan rate of 3 spectra/sec. Abundance dependent accumulation was enabled with a target of 80,000 counts/spectrum. Using auto-MS/MS mode, the ten most abundant precursors per cycle were selected for fragmentation (absolute threshold: 3000 counts; relative threshold: 0.01%). Singly-charged precursors were excluded.

### Analysis of Mass Spectrometric Data

Data were searched using the Spectrum Mill MS Proteomics Workbench (Agilent, Rev B.06.00.201) and the SwissProt human proteome database. For data extraction, spectrum merging was enabled based on precursor selection purity, spectral similarity, retention time (2-minute window), and m/z. Search settings were as follows: instrument = Agilent ESI Q-TOF; precursor mass tolerance = +/- 10 ppm; product ion mass tolerance = +/- 20 ppm; digest = no enzyme. Matches were considered valid if the following thresholds were satisfied: score > 10, percentage scored peak intensity (SPI) > 70%, and rank 1 minus rank 2 (R1-R2) score > 2.5. Spectra that could not be confidently matched to unmodified peptides in the SwissProt database were subjected to a second round of searches using the settings listed above, except oxidized methionine and deamidated asparagine/glutamine were considered as variable modifications and digest was set to AspN (maximum of two missed cleavages). Spectra that remained unmatched according to the validation filters listed above were searched against a custom database containing hypothetical human HIPs (2) using the same settings as in the first search.

### Validation of HIP11 Identification Using Synthetic Peptide and the P-VIS Approach

The HIP11 peptide-spectrum match (PSM) was first evaluated by applying our previously-published criteria (2). A synthetic version of the HIP11 peptide (GenScript, >95% pure) was then used to validate the match by applying the novel **P**SM **V**alidation with **I**nternal **S**tandards (P-VIS) approach recently developed by our group (9). In bottom-up proteomics, proteins from a biological spectrum are digested using a protease such as trypsin. The resultant peptides are analyzed by LC-MS/MS to generate fragmentation spectra. These spectra are then used to search a protein database, generating a large number of PSMs. Traditionally, to confirm that the correct sequence interpretation has been assigned to a particular fragmentation spectrum, the fragmentation spectrum of a synthetic version of the candidate sequence is compared to the fragmentation spectrum of the biological peptide in question using a metric such as the Pearson correlation coefficient. Since two spectra for the same peptide will not be entirely identical, the investigator decides subjectively if the similarity is high enough to indicate that the synthetic peptide and the biological peptide are the same. In the P-VIS approach (9), a set of internal standard peptides is spiked into both the biological sample and the synthetic peptide sample prior to LC-MS/MS analysis. For each internal standard peptide, the fragmentation spectra from the two samples are compared and the Pearson correlation coefficient is calculated. This information is used to determine a prediction interval for the Pearson correlation coefficient of a correct match. A similar process is used to determine if the chromatographic retention time of the biological peptide and synthetic peptide are close enough to indicate that the peptides are the same. PROCAL peptides (JPT Peptide Technologies) were used as internal standard peptides (10). LC-MS/MS analysis was performed as described above, except precursor selection was limited to those masses predicted for the peptides of interest. PSM_validator v1.4 (https://github.com/Delong-Lab/PSM_validator/releases) was used for data analysis with the following settings applied: pre_mz_tol = 20, pro_mz_tol = 20, abund_thresh = 500, PCC_abund_thresh = 10, min_score = 30, min_weighted_score = 25, min_pairs_PCC = 8, min _PCC = 0.7, RTtol = 0.6, min_RT = 10, max_RT = 90, manual_RTdev_thresh = 0, min_intstd = 10, percentile_thresh = 5, ion_type = “b/y”.

## Results

### Response to HIP11 by the E2 CD4 T Cell Clone Is DQ2-Restricted

**Figure 1:**
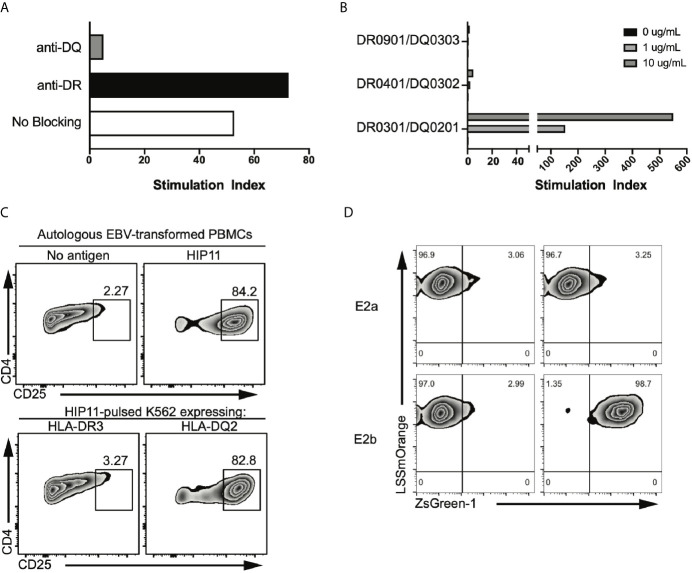
The E2 TCR consisting of the TRAV8-2/8-4 TCRα chain and the TRBV5-4 TCRβ chain recognizes HIP11 in the context of DQ2. **(A)** Peptide-specific proliferation of the E2 T cell clone was assessed using cells from an autologous EBV transformed B cell line as antigen presenting cells and pulsing with 10 µg/mL of the cognate HIP11 peptide in the presence or absence of anti-DR (black bar) or anti-DQ (grey bar) blocking antibody. **(B)** HLA restriction was further defined using cells from partially HLA-matched third-party donors as antigen presenting cells. Irradiated PBMC from each third party donor were pulsed with the cognate HIP11 peptide at concentrations of 0, 1, and 10 µg/mL. Substantial proliferation was observed only in response to the DQA1*05:01-DQB1*02:01 (HLA-DQ2) positive antigen presenting cells, indicating a DQA1*05:01-DQB1*02:01-restricted response. All data are represented as stimulation index (SI) values, calculated by normalizing the proliferation of the T cell clone based on [3H] thymidine incorporation of un-stimulated wells. **(C)** To confirm DQ2 restriction, the E2 T cell clone was cultured in the presence of either an autologous B cell line or K562 lines expressing DR3 or DQ2. After 18h, cells were harvested and stained for CD4 and CD25, and CD25 expression was monitored by flow cytometry. Data is representative of 3 independent experiments. **(D)** Two TCR alpha sequences and one beta sequence were identified from the E2 clone. Each TCR alpha/beta combination, E2a and E2b, was expressed in 5KC T-hybridoma cells with an NFAT-reporter. ZsGreen-1 is expressed upon T cell activation. The E2a and E2b TCR transductants were cultured with the HIP-11 peptide in the presence of K562 cells expressing DQ2 followed by evaluation of ZsGreen-1 expression by flow cytometry.

The HIP11-reactive CD4 T cell clone E2 was isolated from the peripheral blood of a patient with T1D (patient 3196) and in a previous study, we determined that the E2 response to HIP11 was restricted to HLA-DQ (6). We further confirmed these findings using thymidine incorporation as a read-out for T cell proliferation (**Figure 1A**). Since patient 3196 carries one copy of the DQ2 haplotype (DQA1*05:01-DQB1*02:01) and one copy of the DQA1*03:02-DQB1*03:03 haplotype, our goal was to determine the presenting HLA element. Using donor PBMCs partially matched for each haplotype as APCs, we tested the ability of the two haplotypes to stimulate the E2 CD4 T cell clone to HIP11. The E2 T cell clone proliferated when stimulated with the HIP11 peptide insC_20-26_ – insC_1-7_ (SLQPLAL–EAEDLQV) presented by PBMCs homozygous for DQ2, but not when presented by PBMCs homozygous for DQA1*03:02-DQB1*03:03 (**Figure 1B**). As an additional control, we determined that mis-matched PBMCs homozygous for DQ8 (DQA2*0301-DQB2*0302) did not stimulate the E2 T cell clone either at the 1 μM or the 10 μM concentration. To provide additional evidence of the presenting element, we used an artificial antigen-presenting line (K562) devoid of any HLA molecules and transduced with DQ2 or HLA-DRB1*03:01 (DR3) as a negative control (8). Each K562 line (expressing either DQ2 or DR3) was pulsed with the HIP11 peptide and cultured with the E2 T cell clone (**Figure 1C**). After 18h, upregulation of CD25 was assessed by flow cytometry as a measure of T cell activation. The E2 CD4 T cell clone upregulated CD25 in response to HIP11 only when the K562 line was transduced with DQ2, but not DR3, confirming our previous observation that the E2 T cell clone is stimulated by HIP11 in the context of DQ2.

### Identification of TCR Responsible for Recognition of HIP11 by the E2 Clone

TCR sequencing of the E2 CD4 T cell clone revealed that two different TCRα chains – along with a single TCRβ chain – were expressed at the RNA level (6). It has been shown that dual TCRα-expressing T cells can contribute to autoimmunity through different processes, including escape of central tolerance (11) and failure to generate thymically-derived Tregs reactive to self-antigens (12). To determine which TCRα was responsible for the recognition of HIP11, each TCRα chain was expressed individually in combination with the cognate TCRβ chain in a TCR-deficient hybridoma (5KC) engineered with a ZsGreen-1 fluorescent protein gene, under the NFAT promotor (8). Under these conditions, the TCR transductants express ZsGreen-1 when the TCR is engaged with the peptide-MHC complex, enabling monitoring of T cell activation by flow cytometry. The resulting transductants, E2a and E2b (expressing either TRAV6 or TRAV8-2/8-4 respectively), were then challenged with HIP11-pusled artificial APCs (K562 line expressing DQ2). The E2b – but not the E2a – transductant recognized HIP11 in the context of DQ2 (**Figure 1D**), demonstrating that the combination of the TRAV8-2/8-4 TCRα chain with the TRBV5-4 TCRβ chain is responsible for recognizing HIP11.

### Characterization of a HIP11-Specific TCR From the Islets of a T1D Donor

**Figure 2:**
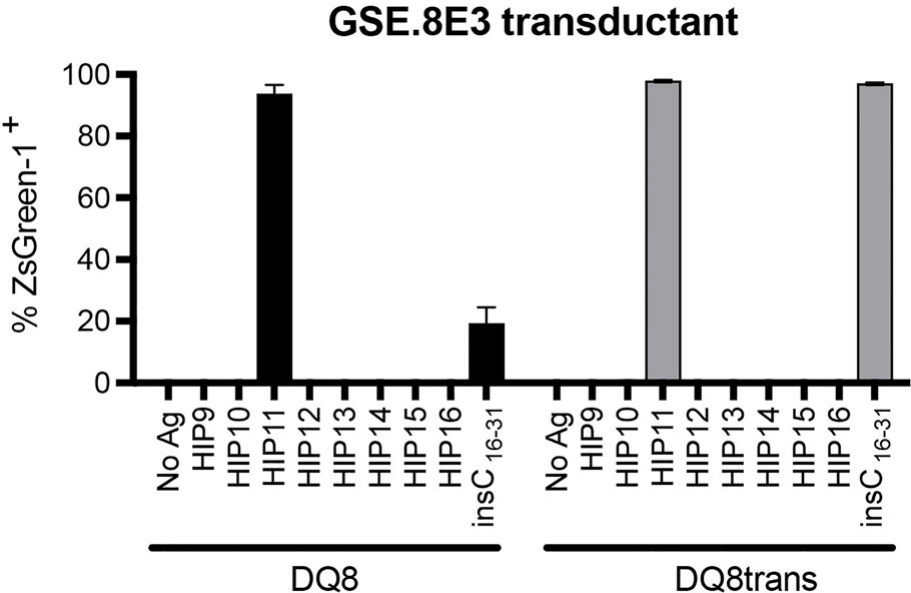
The GSE.8E3 transductant recognizes HIP11 in the context of DQ8 and DQ8-trans. The GSE.8E3 TCR clonotype identified from a CD4 T cell in the islets of a T1D organ donor was expressed in 5KC T-hybridoma cells with an NFAT-reporter. The GSE.8E3 TCR transductant was cultured with or without various HIPs (10 μM) or a native proinsulin peptide (100 μM) in the presence of K562 cells expressing DQ8 (black bars) or DQ8-trans (gray bars). ZsGreen-1 expression was evaluated by flow cytometry. Each result is the mean for three independent experiments. Error bars indicate the standard error of the mean.

**Figure 3:**
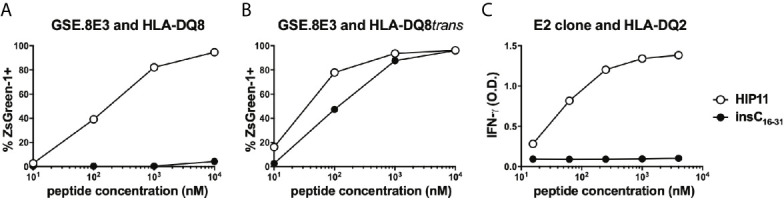
Response of HIP11-reactive T cells to insulin C-peptide. **(A, B)** The GSE.8E3 TCR transductant was cultured with various concentrations of a HIP11 peptide or an insulin C-peptide sequence in the presence of K562 cells expressing DQ8 **(A)** or DQ8*trans* **(B)**. ZsGreen-1 positivity was assessed by flow cytometry as a measure of activation. Data are representative of three independent experiments. **(C)** The E2 T cell clone was cultured with various concentrations of a HIP11 peptide or an insulin C-peptide sequence in the presence of an autologous EBV-transformed B cell line. After 48 hours, supernatants from T cell cultures were harvested and IFN-γ concentrations were measured by ELISA. Results are representative of two independent experiments.

We previously isolated T cell clones and lines from the residual islets of human T1D donors recognizing four different HIPs containing a sequence from the C-peptide linked to insulin A-chain, Islet Amyloid Polypeptide, or Neuropeptide Y (1, 5). Although the E2 clone, which was isolated from the peripheral blood of a T1D patient, is specific for HIP11, isolating a HIP11-reactive T cell clone from the islets – the actual site of autoimmune destruction – would further establish the relevance of HIP11 as an autoantigen in human T1D. Michels et al. previously generated transductants expressing TCRs identified by direct TCR sequencing of islet-infiltrating T cells in organ donors with T1D (13). Several of the CD4 T cell transductants were shown to be reactive to proinsulin peptides. The CD4 T cell transductant GSE.8E3 responded to the peptide insC_17-33_ (GAGSLQPLALEGSLQKR), a region of the C-peptide prone to HIP formation (2). We previously tested reactivity of human PBMCs to eight HIPs containing that region of the C-peptide (6) and found that reactivity to two of them (HIP11 and HIP16) was increased (compared to no antigen) in T1D patients but not in control subjects (6). We hypothesized that post-translational modification of the C-peptide fragment through hybrid peptide formation would generate a more potent antigen for GSE.8E3. Eight HIPs containing the insulin C-peptide fragment insC_20-26_ as the left peptide were tested for antigenicity. The GSE.8E3 transductant strongly reacted to the HIP11 peptide insC_20-26_ – insC_1-7_ (SLQPLAL–EAEDLQV) in the context of both DQ8 and DQ8*trans* (**Figure 2**). When DQ8 was the presenting element, GSE.8E3 did not respond to the native insulin peptide insC_16-31_ (PGAGSLQPLALEGSLQ) (**Figure 3A**) except at very high concentrations (**Figure 2**). GSE.8E3 did respond to insC_16-31_ when presented by DQ8-trans (**Figure 3B**). The E2 clone did not respond to native C-peptide when presented by DQ2 (**Figure 3C**).

### E2 and GSE.8E3 Recognize Different Minimal Epitopes of HIP11

**Figure 4:**
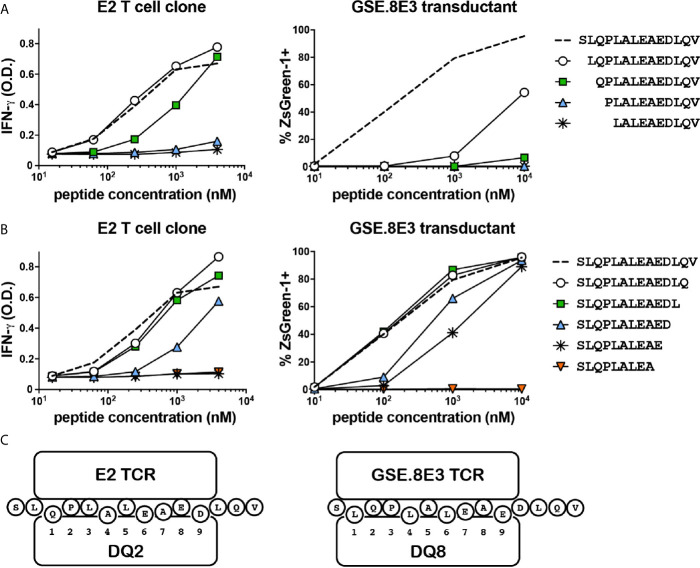
The E2 CD4 T cell clone and the GSE.8E3 TCR transductant recognize different minimal epitopes. N-terminally **(A)** and C-terminally **(B)** truncated HIP11 peptides were tested with E2 and GSE.8E3 for antigenicity. Results are representative of three independent experiments. **(C)** Proposed binding registers for HIP11 in the context of DQ2 or DQ8 when recognized by E2 or GSE.8E3, respectively.

Although the E2 and GSE.8E3 TCRs both recognized HIP11, it was possible that they were specific for different HIP11 epitopes, particularly since recognition by these TCRs was restricted to different HLA molecules. Identifying the precise epitope recognized by each TCR could facilitate future efforts to characterize the immune response to HIP11. For example, it could enable the generation of HLA class II tetramers that allow for detection of different subsets of HIP11-reactive T cells in human subjects. To determine the minimal epitope recognized by the E2 T cell clone and the islet-derived GSE.8E3 transductant, we tested reactivity to HIP11 peptides truncated either on the N- or the C-terminus. Our data show that the response by the E2 T cell clone was completely abrogated when the glutamine on the N-terminus was missing (**Figure 4A**) and when the aspartic acid (D) on the C-terminal region was truncated (**Figure 4B**), indicating that the minimal HIP11 epitope seen by the E2 T cell clone is QPLALEAED. For the GSE.8E3 transductant, our data show that the minimal epitope presented by DQ8 is LQPLALEAE (**Figures 4A, B**). Results were similar when testing the GSE.8E3 response using DQ8*trans* as the presenting element (**Supplementary Figure 1**). Residues flanking these minimal epitopes also seem to contribute to overall activity, possibly as TCR contact residues outside of the peptide binding groove. Based on these data and published data regarding preferred binding residues for DQ2 and DQ8 (14, 15), we propose that HIP11 is presented in two different binding registers depending on whether DQ2 or DQ8 is the presenting element, as shown in **Figure 4C**.

### Design and Validation of an HLA-DQ2/HIP11 Tetramer

**Figure 5:**
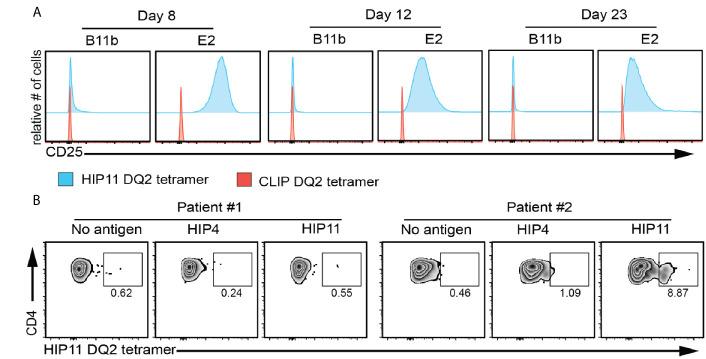
HIP11/DQ2 tetramer stains the E2 T cell clone and PBMCs from a T1D patient. **(A)** The E2 or B11b T cell clones were stimulated with HIP11 or HIP4 respectively and an irradiated autologous EBV-transformed B cell line. T cells were then maintained in culture and harvested at 8, 12 and 23 days post-stimulation and stained with the DQ2/HIP11 tetramer (blue histograms) or DQ2/CLIP tetramer (red histograms), CD4, and a fixable viability dye. Tetramer staining was assessed by flow cytometry by gating on live, CD4^+^ cells. Data are representative of two independent experiments. **(B)** PBMCs from two DQ2+ patients were isolated, stained with CFSE and cultured with medium only, HIP4, or HIP11. After 10 days, cells were harvested and stained with the DQ2/HIP11 tetramer, anti-CD4 and anti-CD25 antibodies, and a fixable viability dye before flow cytometry analysis. Gates were set on live, CD4^+^ CD25^+^ CFSE^low^ events and the percentage of tetramer positive events is reported.

The study of antigen-specific T cells is a high priority in T1D research as CD4 T cells reactive to islet antigens could potentially serve as biomarkers of disease progression in at-risk subjects. While ELISpot analysis can detect cytokine-secreting cells independent of MHC restriction, HLA class II tetramers allow for the detection, enumeration, and phenotyping of antigen-specific T cells directly *ex vivo* (16). Based on the discovery that the response of the HIP11-reactive CD4 T cell clone E2 to HIP11 was DQ2-restricted, we designed an HLA class II tetramer loaded with the HIP11 peptide and tested its ability to stain the E2 T cell clone. As a negative control we used the T cell clone B11b, which is reactive to HIP4, a C-peptide/A-chain hybrid peptide (**Supplementary Figure 2**). While the HIP11-reactive T cell clone stained with the DQ2/HIP11 tetramer, the HIP4-reactive T cell clone B11b did not (**Figure 5A**). Neither of the CD4 T cell clones stained with a negative control DQ2 tetramer loaded with the CLIP peptide (**Figure 5A**). To determine whether antigen stimulation of the T cell clone could improve tetramer binding, the E2 and B11b T cell clones were stimulated with HIP11 or HIP4 respectively in the presence of autologous EBV-transformed PBMCs. T cells were stained with either the HIP11 or the CLIP tetramers 8, 12 and 23 days after stimulation and tetramer staining was monitored by flow cytometry (**Figure 5A**). Our data indicate that the strongest staining is obtained within a week after stimulation. Finally, PBMCs from two DQ2+ T1D patients (with T1D onset less that 2 years) were cultured with medium only or in the presence of HIP4 or HIP11. After 10 days of culture, cells were harvested and stained with the HIP11 tetramer. A substantial population of CD4 T cells present in one of the patients stained with the HIP11/DQ2 tetramer when PBMCs were stimulated with HIP11, but not when stimulated with an irrelevant HIP (**Figure 5B**).

### HIP11 Is Present in Human Islets

**Figure 6:**
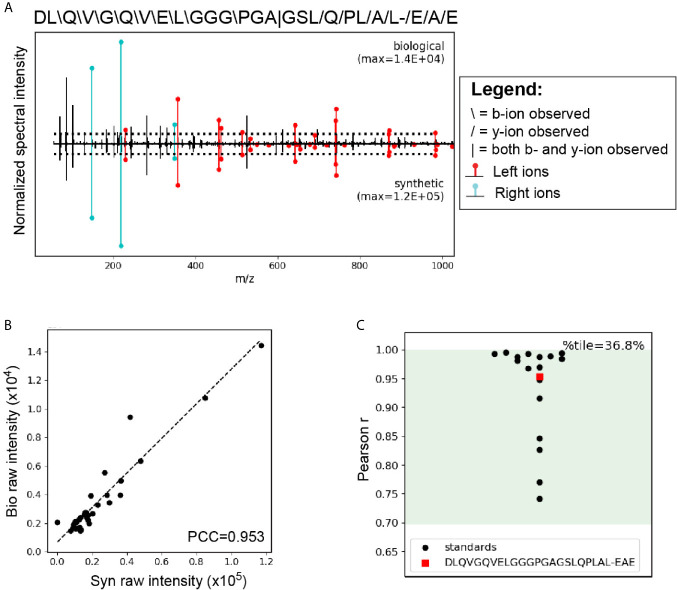
LC-MS/MS analysis using the P-VIS approach confirms that HIP11 is present in human islets. Proteins were extracted from human islets, fractionated by size exclusion chromatography (SEC), and digested with the protease AspN, which cleaves at the N-terminus of aspartic acid (D) residues. Samples were analyzed by LC-MS/MS and data were searched using Agilent Spectrum Mill software. The predicted HIP11 AspN cleavage product DLQVGQVELGGGPGAGSLQPLAL-EAE was identified with high confidence and validated using the P-VIS approach. **(A)** Mirror plot generated by PSM_validator displaying the spectrum from the biological sample (positive y-axis) and the spectrum from the validation sample (negative y-axis). Peak intensities are normalized to the most intense peak in each spectrum. The intensity of the tallest peak in each spectrum is indicated. In the sequence map, “”, “/”, and “|” indicate that the spectrum from the biological sample contained a b-ion, y-ion, or both, respectively, corresponding to fragmentation at a given bond. Horizontal dotted lines indicate the user-defined threshold for consideration of peaks in the Pearson correlation coefficient (PCC) calculation. Ions corresponding to fragmentation of a bond to the left (L ions) or right (R ions) of the hybrid junction are labeled in red and cyan, respectively. **(B)** Peaks that were present above the intensity threshold in at least one of the spectra were used to calculate the Pearson correlation coefficient (PCC) between the two spectra. For each peak, the intensity in the biological peptide spectrum (bio raw intensity) and the intensity in the spectrum for the synthetic peptide (syn raw intensity) were paired, and the pairs for all peaks were plotted. Calculation of the PCC for these values indicated that the spectra were highly correlated (PCC = 0.953). **(C)** Comparison of the PCC for the HIP11 match to the distribution of PCCs for internal standard peptides (ISPs). Data for the ISPs passed the D’Agostino-Pearson omnibus normality test (p = 0.229; p > 0.05 indicates that the null hypothesis cannot be rejected and the data can be treated as normal). Green shading indicates the 95% prediction interval based on one-tailed analysis of Fisher’s z-transformed PCC values. The PCC comparing the biological spectrum and the validation spectrum is shown (red square), and the percentile (%tile) is reported.

A critical step in confirming that HIP11 is a natural antigen in human T1D was to identify HIP11 in human islets by mass spectrometry (LC-MS/MS). Proteins were isolated from non-diabetic human donor islets and digested with the protease AspN to generate peptides suitable for mass spectrometric analysis. Resultant peptides were then analyzed by LC-MS/MS, and data were searched against a custom database to find peptides describing the HIP11 hybrid junction. Our analysis identified the peptide insC_4-26_ – insC_1-3_ (DLQVGQVELGGGPGAGSLQPLAL-EAE) with high confidence (**Figure 6A** and **Supplementary Table 2**). This peptide is the predicted AspN cleavage product of HIP11. We previously established a set of rigorous criteria to confidently identify HIPs by mass spectrometry (2), and the putative HIP11 match satisfied all of these criteria (**Supplementary Table 3**). To further confirm the validity of the mass spectrometry-based HIP11 identification, we applied a novel approach – **P**eptide-spectrum match **V**alidation with **I**nternal **S**tandards (P-VIS) – recently developed by our group (9). Using this approach, the fragmentation spectrum of the peptide found in human islets was found to be highly similar to the spectrum of synthetic insC_4-26_ – insC_1-3_ peptide (**Figure 6A**), with a Pearson correlation coefficient of 0.953 (**Figure 6B**). Based on the data for internal standard peptides, this degree of correlation indicated that the peptide found in human islets was indeed the HIP11 peptide insC_4-26_ – insC_1-3_ (**Figure 6C**). This finding was confirmed by P-VIS chromatographic retention time analysis (**Supplementary Figure 3**).

## Discussion

The discovery of HIPs in human islets (2) and the detection and isolation of HIP-reactive CD4 T cells from the peripheral blood and islets of individuals with T1D (1, 5–7) indicate a role for HIPs as autoantigens in T1D. Here, we used LC-MS/MS analysis to report for the first time that HIP11, a C-peptide/C-peptide HIP, is present in human islets. We determined the TCR Vα and Vβ usage and the HLA class II restriction of the HIP11-reactive E2 clone previously isolated from the peripheral blood of a T1D donor. We also demonstrated that HIP11 is a potent epitope for a proinsulin-specific T cell receptor (GSE.8E3) identified by analysis of islet-infiltrating T cells from a T1D organ donor (13). Notably, we found that GSE.8E3 and the E2 clone each recognize a different minimal epitope of HIP11 and recognition by these clones was restricted to HLA-DQ8/HLA-DQ8*trans* or HLA-DQ2, respectively. HLA-DQ8 and HLA-DQ8*trans* consist of the same β chain paired with different α chains. A previous study (17) demonstrated that CD4 T cell clones (isolated from T1D donors and specific for immunodominant islet epitopes) could recognize their cognate peptide epitopes presented by either DQ8 or DQ8*trans*, with presentation by DQ8*trans* eliciting a stronger response. The DQ8*trans* α chain lacks the Arg52 residue present in the DQ8 α chain. The authors demonstrated that the absence of Arg52α led to differences in the orientation of TCR contact residues in the bound peptide, resulting in a higher affinity between the TCR and the peptide-DQ8*trans* complex (17). The GSE.8E3 transfectoma was more sensitive to N-terminal and C-terminal truncations of the HIP11 peptide SLQPLAL-EAEDLQV when DQ8 was the presenting element, also suggesting possible differences in the orientation of crucial TCR contacts when either DQ8 or *DQ8trans* was the presenting element. However, structural characterization, such as by X-ray crystallography, would be needed for definitive characterization of the interactions between the TCR, the peptide, and the different HLA molecules.

As more than 73% of all patients with T1D carry either HLA-DQ8 (DQA2*0301-DQB2*0302), HLA-DQ2 (DQA1*0501-DQB1*0201), or both (18), presentation by two different HLA molecules strongly associated with disease susceptibility further supports the case for HIP11 as a relevant autoantigen in T1D. Of note, individuals that carry both DQ2 and DQ8 alleles have a higher risk of developing T1D (19) and investigation of the relevance of HIP11 in this population is currently underway in our lab. Like I-A^g7^ in the NOD mouse model of autoimmune diabetes, position 57 of the β-chain of HLA-DQ8 and HLA-DQ2 has a non-aspartic acid (D) amino acid polymorphism that favors the presentation of an acidic amino acid in p9 of the binding groove (20). While there are clear amino acid preferences for binding to either molecule in pockets 1, 4, 6, 7 and 9 (14, 15, 21), the possibility of forming HLA trans dimers offers additional possibilities for peptide binding while maintaining the β57 non-D amino acid (22).

Formation of HIPs in pancreatic β-cells is likely the result of the cellular environment where unique peptides, such as the insulin C-peptide, are present at high local concentrations inside secretory granules. This could lead to the formation of specific HIPs that do not form in the thymus. Thus, central tolerance to these HIPs may not be established, allowing for the development of autoreactivity to HIPs in the periphery. Cross-reactivity of HIP-reactive T cells to unmodified peptides such as C-peptide may then lead to a break in tolerance to these unmodified insulin peptides, potentially resulting in a more robust autoimmune response. We observed, for example, that the GSE.8E3 transductant, which responded to HIP11 in the context of either HLA-DQ8 or HLA-DQ8*trans*, also responded to native C-peptide but only when presented by DQ8*trans* and not by DQ8. One could speculate that the ability of HLA-DQ8*trans* to present both a HIP and an unmodified insulin peptide to an autoreactive T cell clone in a stimulatory fashion suggests a possible mechanism in which carrying both the HLA-DQ2 and HLA-DQ8 alleles increases the risk of developing T1D by facilitating T cell cross reactivity to HIPs and unmodified peptides. It should be noted that HIP11 could be found in the islets of a non-T1D organ donor, suggesting that HIP formation is not limited to humans with autoimmunity. Similarly, insulin has long been recognized as an autoantigen in T1D, even though it is expressed in all individuals. Elucidating how various factors such as HIP formation and HLA genotype act in concert to promote disease development could be key to future progress in T1D research.

The former gold standard for validating mass spectrometry-based peptide identifications was to compare the fragmentation spectrum and chromatographic retention time for a synthetic version of a putative peptide sequence to those observed for the biological peptide. Our P-VIS approach (9) adds rigor and objectivity to this process by using comparisons for internal standard peptides to evaluate if the observed degree of similarity between the biological and synthetic peptides is high enough to indicate a correct match. Although we confidently identified HIP11 in human islets, the observed fragment provides limited information about the origin and mechanism of formation of the biological peptide since human islet proteins were digested with the AspN protease prior to LC-MS/MS analysis. Could HIP11 be the product of intramolecular transpeptidation and therefore the generation of a cis-hybrid? In this scenario, a protease would remove the five C-terminal residues from a C-peptide molecule and fuse the N-terminus of the same molecule to the newly-formed C-terminus, thereby generating a cyclic peptide. Cleavage of this cyclic peptide by a protease or the proteasome complex could then generate a linear HIP11 peptide suitable for presentation by MHC molecules. Alternatively, HIP11 could be formed by intermolecular transpeptidation (formation of a trans-hybrid), in which one C-peptide molecule is cleaved, generating the insC_1-26_ fragment, and the N-terminus of a separate C-peptide molecule is fused to the C-terminus of the insC_1-26_ fragment. Various other details of how HIP11 is formed are also unknown including for example, the specific enzyme responsible for formation of HIP11 in human islets.

In combination with our previous work, our results provide a thorough analysis of a disease-relevant HIP in human T1D, including identification of the HIP in islets, investigation of CD4 T cell responses, and molecular characterization of epitope recognition. The validation of an HLA-DQ2/HIP11 tetramer opens the path for further characterization of the role of HIP11-reactive T cells in T1D. To our knowledge, this is one of the first DQ2 tetramers loaded with a T1D disease-relevant antigen. This work provides a precedent for in-depth characterization of responses to HIPs as important components of the autoimmune response in T1D.

## Data Availability Statement

The original contributions presented in the study are included in the article/**Supplementary Material**. The data presented in the study are included in the article figures, tables, and supplementary materials. The T cell receptor alpha and beta chain sequences and corresponding epitope data were submitted to the immune epitope database (https://www.iedb.org/home_v3.php) and can be accessed as submission ID 1000868. Further inquiries about the data can be directed to the corresponding author.

## Ethics Statement

The studies involving human participants were reviewed and approved by the Colorado Multiple Institutional Review Board. Written informed consent to participate in this study was provided by the participants’ legal guardian/next of kin.

## Author Contributions

RB, TW, MN, and EJ designed experimental work. AH, RB, TW, MD, LL, RP, and MN performed experimental work. RB, TW, KH, and TD wrote and edited the manuscript. All authors contributed to the article and approved the submitted version.

## Funding

This study was supported by the American Diabetes Association (ADA) Pathway to stop Diabetes 1-15-ACE-14 (TD), National Institutes of Health (NIH) research grants R21AI133059 (RB), R01 AI146202-01A1 (RB), R01DK081166 (KH), R01DK119529-02 (TD), R01DK099317 (MN), R01DK032083 (MN), Juvenile Diabetes Research Foundation grants 2-SRA-2016-226-S-B (TD), 1-SRA-2020-911-A-N (MN), and PDF-2019-746-A-N (TW).

## Conflict of Interest

The authors declare that the research was conducted in the absence of any commercial or financial relationships that could be construed as a potential conflict of interest.

## References

[1] Delong T Wiles TA Baker RL Bradley B Barbour G Reisdorph R. Pathogenic CD4 T Cells in Type 1 Diabetes Recognize Epitopes Formed by Peptide Fusion. Science (80- ) (2016) 351:711–4. 10.1126/science.aad2791 PMC4884646 26912858

[2] Wiles TA Powell R Michel CR Scott Beard K Hohenstein A Bradley B. Identification of Hybrid Insulin Peptides (HIPs) in Mouse and Human Islets by Mass Spectrometry. J Proteome Res (2019) 18:814–25. 10.1021/acs.jproteome.8b00875 PMC7597855 30585061

[3] Wan X Vomund AN Peterson OJ Chervonsky AV Lichti CF Unanue ER. The MHC-II Peptidome of Pancreatic Islets Identifies Key Features of Autoimmune Peptides. Nat Immunol (2020) 21:455–63. 10.1038/s41590-020-0623-7 PMC7117798 32152506

[4] Baker RL Jamison BL Wiles TA Lindsay RS Barbour G Bradley B. CD4 T Cells Reactive to Hybrid Insulin Peptides Are Indicators of Disease Activity in the NOD Mouse. Diabetes (2018) 67:1836–46. 10.2337/db18-0200 PMC6110316 29976617

[5] Babon JAB DeNicola ME Blodgett DM Crèvecoeur I Buttrick TS Maehr R. Analysis of Self-Antigen Specificity of Islet-Infiltrating T Cells From Human Donors With Type 1 Diabetes. Nat Med (2016) 22:1482–7. 10.1038/nm.4203 PMC5140746 27798614

[6] Baker RL Rihanek M Hohenstein AC Nakayama M Michels A Gottlieb PA. Hybrid Insulin Peptides Are Autoantigens in Type 1 Diabetes. Diabetes (2019) 68:1830–40. 10.2337/db19-0128 PMC6702640 31175101

[7] Arribas-Layton D Guyer P Delong T Dang M Chow IT Speake C. Hybrid Insulin Peptides Are Recognized by Human T Cells in the Context of Drb1*04:01. Diabetes (2020) 69:1492–502. 10.2337/db19-0620 PMC7306133 32291282

[8] Mann SE Zhou Z Landry LG Anderson AM Alkanani AK Fischer J. Multiplex T Cell Stimulation Assay Utilizing a T Cell Activation Reporter-Based Detection System. Front Immunol (2020) 11. 10.3389/fimmu.2020.00633 PMC7160884 32328071

[9] Wiles TA Saba LM Delong T. Peptide–Spectrum Match Validation With Internal Standards (P–VIS): Internally-Controlled Validation of Mass Spectrometry-Based Peptide Identifications. J Proteome Res (2020) 20(1):236–49. 10.1021/acs.jproteome.0c00355 PMC7775876 32924495

[10] Zolg DP Wilhelm M Yu P Knaute T Zerweck J Wenschuh H. PROCAL: A Set of 40 Peptide Standards for Retention Time Indexing, Column Performance Monitoring, and Collision Energy Calibration. Proteomics (2017) 17. 10.1002/pmic.201700263 28872757

[11] Ji Q Perchellet A Goverman JM. Viral Infection Triggers Central Nervous System Autoimmunity Via Activation of CD8 + T Cells Expressing Dual TCRs. Nat Immunol (2010) 11:628–34. 10.1038/ni.1888 PMC2900379 20526343

[12] Schuldt NJ Auger JL Spanier JA Martinov T Breed ER Fife BT. Cutting Edge: Dual TCRα Expression Poses an Autoimmune Hazard by Limiting Regulatory T Cell Generation. J Immunol (2017) 199:33–8. 10.4049/jimmunol.1700406 PMC5501482 28539428

[13] Michels AW Landry LG McDaniel KA Yu L Campbell-Thompson M Kwok WW. Islet-Derived CD4 T Cells Targeting Proinsulin in Human Autoimmune Diabetes. Diabetes (2017) 66:722–34. 10.2337/db16-1025 PMC5319719 27920090

[14] Vartdal F Johansen BH Friede T Thorpe CJ Stevanović S Eriksen JE. The Peptide Binding Motif of the Disease Associated HLA-DQ (α 1(*) 0501, β 1(*) 0201) Molecule. Eur J Immunol (1996) 26:2764–72. 10.1002/eji.1830261132 8921967

[15] Chang KY Unanue ER. Prediction of HLA-DQ8 Cell Peptidome Using a Computational Program and Its Relationship to Autoreactive T Cells. Int Immunol (2009) 21:705–13. 10.1093/intimm/dxp039 PMC2686615 19461125

[16] Nepom GT. MHC Class II Tetramers. J Immunol (2012) 188:2477–82. 10.4049/jimmunol.1102398 PMC3297979 22389204

[17] Chow IT Gates TJ Papadopoulos GK Moustakas AK Kolawole EM Notturno RJ. Discriminative T Cell Recognition of Cross-Reactive Islet-Antigens Is Associated With HLA-DQ8 Transdimer–Mediated Autoimmune Diabetes. Sci Adv (2019) 5:eaaw9336. 10.1126/sciadv.aaw9336 PMC6703875 31457096

[18] Erlich H Valdes AM Noble J Carlson JA Varney M Concannon P. HLA DR-DQ Haplotypes and Genotypes and Type 1 Diabetes Risk Analysis of the Type 1 Diabetes Genetics Consortium Families. Diabetes (2008) 57:1084–92. 10.2337/db07-1331 PMC4103420 18252895

[19] Pociot F McDermott MF. Genetics of Type 1 Diabetes Mellitus. Genes Immun (2002) 3:235–49. 10.1038/sj.gene.6363875 12140742

[20] Jones EY Fugger L Strominger JL Siebold C. MHC Class II Proteins and Disease: A Structural Perspective. Nat Rev Immunol (2006) 6:271–82. 10.1038/nri1805 16557259

[21] Van De Wal Y Kooy YMC Drijfhout JW Amons R Koning F. Peptide Binding Characteristics of the Coeliac Disease Associated DQ(α1(*)0501, β1(*)0201) Molecule. Immunogenetics (1996) 44:246–53. 10.1007/BF02602553 8753854

[22] Van Lummel M Van Veelen PA De Ru AH Pool J Nikolic T Laban S. Discovery of a Selective Islet Peptidome Presented by the Highest-Risk HLA-DQ8trans Molecule. Diabetes (2016) 65:732–41. 10.2337/db15-1031 26718497

