# Supplementary material for: Characterization of Human CD4 T Cells Specific for a C-Peptide/C-Peptide Hybrid Insulin Peptide
Source: Front Immunol. 2021 May 25;12:668680. doi: 10.3389/fimmu.2021.668680 (PMC8185328; doi:10.3389/fimmu.2021.668680)
Supplement: Supplementary file 1 [file DataSheet_1.pdf]

Supplementary Material

1.1 Supplementary Figures

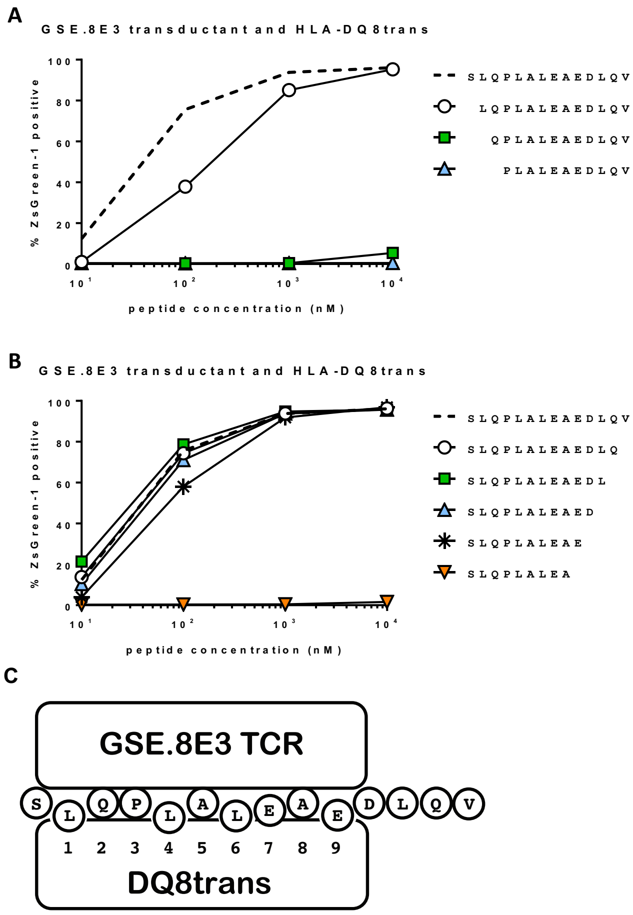

Supplementary Figure 1. Defining the minimal HLA-DQ8*trans*-restricted epitope for the GSE.8E3 TCR transductant. N-terminally (**A**) and C-terminally (**B**) truncated HIP11 peptides were tested with GSE.8E3 for antigenicity when presented by HLA-DQ8*trans*. Results are representative of three independent experiments. **C.** Proposed binding register for HIP11 in the context of DQ8trans when recognized by GSE.8E3.

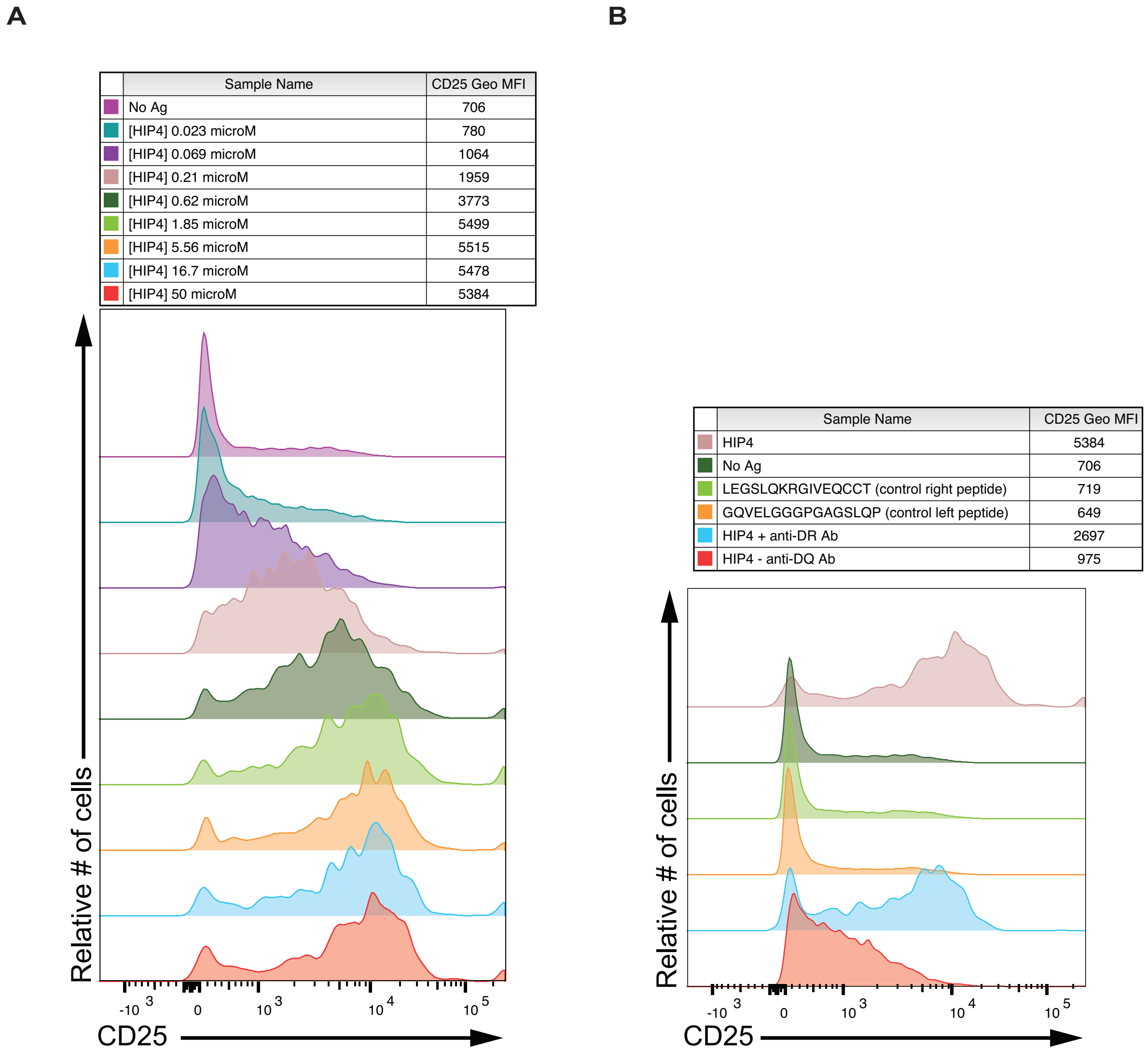

Supplementary Figure 2. Characterization of the HIP4-reactive T cell clone B11b. The B11b T cell clone was isolated from a T1D patient using previously described methodologies (6). Briefly, PBMCs were labeled with CFSE and cultured in AIM V medium containing the HIP4 peptides and normal human AB serum. After 7 days, $CFSE^{dim}$, $CD25^{hi}$, live cells were index sorted into a round-bottom 96-well plate using an Astrios EQ cell sorter (Beckman Coulter) at one cell per well. Wells were supplemented with recombinant human IL-2 (20 U/ml), IL-4 (5 ng/ml), anti-CD3 (30 ng/ml), irradiated PBMCs (mixed from two different donors; 1 x 10^6/ml), and irradiated Priess cells (1 x 10^6/ml). Wells were regularly scored for growth by visual inspection and cells were expanded using IL-2 and IL-4. Antigen-specific T cell clones were selected for further expansion and TCR sequencing. **A.** The T cell clone B11b was challenged with an irradiated autologous EBV-transformed B cell line and various concentrations of HIP4. **B.** HLA restriction was determined using anti-DR or anti-DQ antibodies. Data also show that the T cell clone B11b does not react to right and left control peptides. **A & B.** After 24 hours, cells were harvested and stained for CD4, CD25, and viability. CD25 expression was assessed by flow cytometry and Geometric Mean Fluorescence Intensity (Geo MFI) is reported for each condition. Data are representative of three independent experiments.

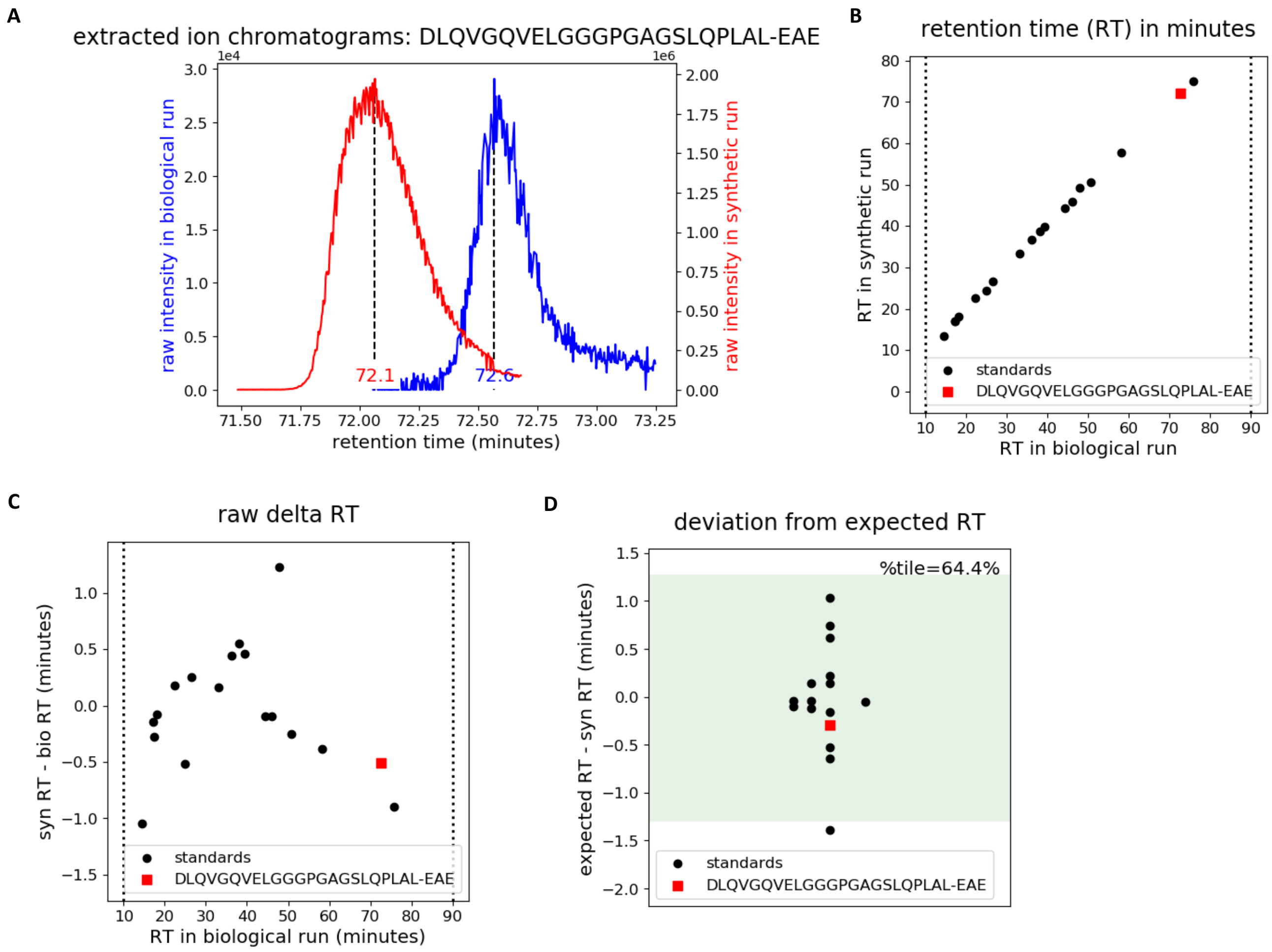

Supplementary Figure 3. Chromatographic retention time analysis using the P-VIS approach confirms that HIP11 is present in human islets. The P-VIS approach was used to compare the putative HIP11 peptide DLQVGQVELGGGPGAGSLQPLAL-EAE observed in a human islet sample to a synthetic version of the proposed peptide. Plots automatically generated by PSM_validator following chromatographic retention time (RT) analysis are shown. **A.** Extracted ion chromatogram for the peptide in the biological sample run and the peptide in the validation sample run. **B.** RT for each of the ISPs between the two sample runs (black circles) and the RT of the biological peptide and the validation peptide (red square). **C.** Difference in chromatographic RT (syn RT – bio RT) for each of the ISPs between the two sample runs (black circles) and the RT difference between the biological peptide and the validation peptide (red square). **D.** A linear spline model was used to align RTs in the biological run with the validation sample run. The difference between the adjusted RT for the biological run (expected RT) and the RT in the validation run (syn RT) is plotted for each ISP (black circles). Data passed the D'Agostino-Pearson omnibus normality test ($p = 0.296$). Green shading indicates the 95% prediction interval based on two-tailed analysis. The difference between the adjusted RT for the biological peptide and the RT for the validation peptide is also shown (red square), and the percentile (%tile) is reported.

1.2 Supplementary Tables

SUPPLEMENTARY TABLE I: SYNTHETIC PEPTIDES USED IN STUDY*

peptide name	HIP?	HIP name	left peptide	right peptide	peptide sequence
insC$_{8\text{-}15}$ – insA$_{1\text{-}7}$	Yes	HIP4	insulin C-peptide	insulin A-chain	GQVELGGG-GIVEQCC
insC$_{20\text{-}26}$ – CgA$_{342\text{-}348}$	Yes	HIP9	insulin C-peptide	chromogranin A	SLQPLAL-WSKMDQL
insC$_{20\text{-}26}$ – CgA$_{358\text{-}364}$	Yes	HIP10	insulin C-peptide	chromogranin A	SLQPLAL-LEGQEEE
insC$_{4\text{-}26}$ – insC$_{1\text{-}3}$	Yes	HIP11	insulin C-peptide	insulin C-peptide	DLQVGQVELGGGPGAGSLQPLAL-EAE
insC$_{20\text{-}26}$ – insC$_{1\text{-}7}$	Yes	HIP11	insulin C-peptide	insulin C-peptide	SLQPLAL-EAEDLQV
insC$_{20\text{-}26}$ – insA$_{1\text{-}7}$	Yes	HIP12	insulin C-peptide	insulin A-chain	SLQPLAL-GIVEQCC
insC$_{20\text{-}26}$ – IAPP$_{23\text{-}29}$	Yes	HIP13	insulin C-peptide	islet amyloid polypeptide	SLQPLAL-TPIESHQ
insC$_{20\text{-}26}$ – IAPP$_{74\text{-}80}$	Yes	HIP14	insulin C-peptide	islet amyloid polypeptide	SLQPLAL-NAVEVLK
insC$_{20\text{-}26}$ – Scg1$_{440\text{-}446}$	Yes	HIP15	insulin C-peptide	secretogranin 1	SLQPLAL-FLGEGHH
insC$_{20\text{-}26}$ – NPY$_{68\text{-}74}$	Yes	HIP16	insulin C-peptide	neuropeptide Y	SLQPLAL-SSPETLI
insC$_{16\text{-}31}$	No	N/A	insulin C-peptide	N/A	PGAGSLQPLALEGSLQ

*Numbering of amino acid residues is based on their position in the pre-proprotein sequence (includes signal peptide). Hyphens indicate a hybrid peptide junction.

SUPPLEMENTARY TABLE II: OBSERVED B- AND Y-IONS IN FRAGMENTATION SPECTRUM FOR HIP11 PEPTIDE IN HUMAN ISLETS*

	predicted m/z	obz m/z	intensity		predicted m/z	obz m/z	intensity
**b1**	116.0342			**y25**	2390.2562		
**b2**	229.1183	229.1166	1987	**y24**	2277.1721		
**b3**	357.1769	357.1749	6371	**y23**	2149.1135		
**b4**	456.2453	456.2433	3438	**y22**	2050.0451		
**b5**	513.2667	513.2650	1706	**y21**	1993.0237		
**b6**	641.3253	641.3217	2687	**y20**	1864.9651		
**b7**	740.3937	740.3908	3286	**y19**	1765.8967		
**b8**	869.4363	869.4312	1453	**y18**	1636.8541		
**b9**	982.5204	982.5142	1553	**y17**	1523.7700		
**b10**	1039.5419			**y16**	1466.7486		
**b11**	1096.5633			**y15**	1409.7271		
**b12**	1153.5848	1153.5736	1139	**y14**	1352.7056		
**b13**	1250.6375			**y13**	1255.6529		
**b14**	1307.6590			**y12**	1198.6314		
**b15**	1378.6961			**y11**	1127.5943	1127.5854	845
**b16**	1435.7176			**y10**	1070.5728		
**b17**	1522.7496			**y9**	983.5408		
**b18**	1635.8337			**y8**	870.4567	870.4485	1909
**b19**	1763.8923			**y7**	742.3981	742.3945	4961
**b20**	1860.9450			**y6**	645.3454		
**b21**	1974.0291			**y5**	532.2613	532.2596	783
**b22**	2045.0662			**y4**	461.2242	461.2209	2233
**b23**	2158.1503			**y3**	348.1401	348.1400	2767
**b24**	2287.1929			**y2**	219.0975	219.0968	14426
**b25**	2358.2300			**y1**	148.0604	148.0596	10738

*Calculated monoisotopic mass-to-charge ratio (predicted m/z) for each possible singly-charged b-ion and y-ion for the sequence DLQVGQVELGGGPGAGSLQPLAL-EAE. The mass-to-charge ratio (obs m/z) and raw intensity for each observed peak matched to a predicted b- or y-ion is also provided. All observed m/z values are within 10 ppm of the predicted values. Results are representative of two independent experiments using different islet aliquots from the same donor.

SUPPLEMENTARY TABLE III: INITIAL VALIDATION OF HIP11 PEPTIDE-SPECTRUM MATCH (PSM)*

Rule	Description	Result	Details
**1**	Spectrum can not be confidently matched to a modified or unmodified peptide in a traditional protein database.	PASSED	For best match in traditional protein database:<br>SPI = 59.7%; score = 7.93; R1-R2 score = 3.85
**2**	Spectrum matches unambiguously and with high confidence to a peptide in the HIP database.	PASSED	For HIP match:<br>SPI = 92.6%; score = 18.97; R1-R2 score = 8.69
**3**	Peptide match corresponds to the proteolytic digest performed in sample preparation.	PASSED	Matches predicted digest with no missed cleavages
**4**	The left peptide and right peptide regions each contain at least two amino acid residues.	PASSED	23 residues in left peptide, 3 in right
**5**	The spectrum contains sufficient b-/y-ion coverage of both the left and right peptide region of the HIP.	PASSED	10 ions covering left peptide, 3 covering right
**6**	The fragmentation spectrum for the endogenous peptide is highly similar to the spectrum obtained upon fragmentation of a synthetic version of the proposed HIP.	PASSED	PCC = 0.953; percentile = 36.8%
**7**	The endogenous peptide and the synthetic HIP reference peptide elute at the same time by chromatography.	PASSED	delta RT = -0.297; percentile = 64.4%

*Scored Peak Intensity (SPI) is the percentage of the total ion intensity in the fragmentation spectrum that can be matched to predicted ions. Score indicates the number of peaks (out of the 25 most abundant peaks in the spectrum) that can be matched to predicted ions; different ion types are weighted differently. R1-R2 score is the difference between the score for the best and second-best match for a given spectrum. We require SPI > 70%, score > 10, and R1-R2 score > 2.5. Results for Rule 5 indicate the number of bonds to the left of the hybrid junction and to the right of the hybrid junction that are covered by b- and/or y-ions. An ion corresponding to fragmentation at the junction bond is counted towards both the left and right peptide coverage. Pearson Correlation Coefficient (PCC), delta RT, and percentiles were calculated using PSM_validator. Delta RT refers to the adjusted value ("expected RT minus syn RT") reported by PSM_validator and calculated using a linear spline model of retention time (RT) variation between the two sample runs.
